# Aerodynamics of a highly irregular body at transonic speeds—Analysis of STRATOS flight data

**DOI:** 10.1371/journal.pone.0187798

**Published:** 2017-12-07

**Authors:** Markus Guerster, Ulrich Walter

**Affiliations:** Institute of Astronautics, Technical University of Munich, Garching, Germany; Universitat Zurich, SWITZERLAND

## Abstract

In this paper, we analyze the trajectory and body attitude data of Felix Baumgartner’s supersonic free fall through the atmosphere on October 14, 2012. As one of us (UW) was scientific advisor to the Red Bull Stratos team, the analysis is based on true body data (body mass, wetted pressure suit surface area) and actual atmospheric data from weather balloon measurements. We also present a fully developed theoretical analysis and solution of atmospheric free fall. By matching the flight data against this solution, we are able to derive and track the drag coefficient *C*_*D*_ from the subsonic to the transonic and supersonic regime, and back again. Although the subsonic drag coefficient is the expected *C*_*D*_ = 0.60 ± 0.05, surprisingly the transonic compressibility drag coefficient is only 19% of the expected value. We provide a plausible explanation for this unexpected result.

## I. Introduction

On October 14, 2012, and as part of the Red Bull Stratos Mission [1], Felix Baumgartner performed a record-breaking supersonic free fall from the stratosphere. Other than from earlier jumps, namely by Joe Kittinger in 1960 [2] and a supersonic jump by Alan Eustace on October 24, 2014 [3], only for Felix’ extensive flight data are available. It is the objective of this paper to understand the physics of the free fall, in particular the aerodynamic behavior in the transonic regime.

The first analysis of a high-altitude free fall dates back to 1996, when Mohazzabi and Shea [4] were able to analytically solve the equation of motion for *v*(*h*) by power series including the atmospheric friction term and a standard barometric law. From a linear approximation of an implicit solution they were able to roughly analyze Kittinger’s jump confirming his key achievements. However, owing to the linear approximations made, the result becomes less reliable for jumps with extensive free fall segments as with Felix Baumgartner’s and Alan Eustace’s jumps. In addition, the power series approximation works only with a strict barometric law and a constant aerodynamic friction term.

In 2010 Benacka [5] revised Mohazzabi’s high-altitude free fall analysis by taking into account a linear temperature gradient as about actual in the troposphere. For a drag coefficient that does not depend on the Mach number he was able to solve the equation of motion analytically by power series, again for *v*(*h*). Later in 2011 Vadim et al. [6] and 2014 Rygalov [7] published two papers. The earlier deals with the theoretical analysis of the maximal drag deceleration in free fall and the corresponding altitude, whereas the latter shows that an emergency egress at an altitude around 100 km can be survived and it provides a relation between the initial altitude and the altitude of the transonic transition. His analysis is based on a drag coefficient independent on the Mach number and on a temperature-independent barometric law.

J. M Colino and A. J Barbero in 2013 [8] published a course quantitative data analysis based on a spreadsheet and intended as an introductory physics course of Felix’ free fall. Although they use a similar approach as described in our paper, they do not have precise data and use only a standard atmosphere model to derive *v*(*h*) and *v*(*t*). Therefore they were not able to extract the key aerodynamic parameter, the drag coefficient *C*_*D*_, from the computed product *C*_*D*_*A*_⊥_, with *A*_⊥_ being the wetted surface area.

Since the insight into the physics of the supersonic jump is gained only through a well-defined drag coefficient *C*_*D*_, a full-fledged numerical investigation with exact data is needed, which is the objective of this paper.

## II. Theoretical analysis

Let *h* be the altitude of the skydiver above ground. We define the downwards velocity *v* to be positive, i.e. *dh* = −*v*⋅*dt*. Then the equation of motion of a body with mass *m* subject to gravitational and aerodynamic forces is known to be

$$\frac{dv}{dt}=g\left(h,\varphi \right)-\frac{1}{2}C_{D}\left(Ma,Re\right)\rho \left(h,T\right)\frac{A_{⊥}}{m}v^{2}$$ (1)

where *C*_*D*_ is the aerodynamic drag coefficient, including the pressure drag, skin friction drag and interference drag. The coefficient quite generally depends on the type of flow (laminar or turbulent) and hence on the Mach number *Ma* and Reynolds number *Re*. We can safely neglect a Knudsen number dependency because at stratospheric altitudes we are in the continuum flow regime. The quantity *ρ* is the atmospheric density depending on altitude and temperature *T*. *A*_⊥_ is the aerodynamically wetted pressure suit area depending on the angle of attack *α* as defined later. We assume *g* to be the gravity of Earth and not Earth’s gravitational acceleration. This is because Earth’s gravity results jointly from Earth’s gravitational force plus the centrifugal force due to the rotation of the Earth and therefore is dependent on the altitude *h* and geographical latitude *φ*. Because of Earth’s atmosphere up to roughly 100 km altitude on average co-rotates with Earth’s surface, we have to take centrifugal forces into account.

We first define the *instantaneous terminal velocity*

$$\frac{1}{v_{t}^{2}}≔\frac{1}{2}C_{D}\rho \frac{A_{⊥}}{mg}$$ (2)

With this the acceleration from Eq (1) can be written as

$$\frac{dv}{dt}=g\left(1-\frac{v^{2}}{v_{t}^{2}}\right)$$ (3)

If *v*_*t*_ would be independent of altitude and velocity, the acceleration would cease at *v* = *v*_*t*_. So, *v*_*t*_ is the terminal velocity at instantaneous flight conditions.

### Analytical solutions for *v*_*t*_ = *const*

Let *v*_0_ = *v*(*t*_0_ = 0) and *h*_0_ = *h*(*t*_0_ = 0). If *v*_*t*_ is considered constant, then Eq (3) can be integrated directly

$$\int_{v_{0}}^{v}\frac{dv}{1-v^{2}/v_{t}^{2}}=\frac{1}{2}v_{t}\ln\frac{\left(v_{t}+v\right)\left(v_{t}-v_{0}\right)}{\left(v_{t}-v\right)\left(v_{t}+v_{0}\right)}=g\int_{0}^{t}dt=gt$$

We finally get

$$\frac{v_{t}+v}{v_{t}-v}=\frac{v_{t}+v_{0}}{v_{t}-v_{0}}\exp\left(\frac{2gt}{v_{t}}\right)$$

or equivalently

$$v\left(t\right)=v_{t}\frac{v_{t}+v_{0}-\left(v_{t}-v_{0}\right)\exp\left(-2gt/v_{t}\right)}{v_{t}+v_{0}+\left(v_{t}-v_{0}\right)\exp\left(-2gt/v_{t}\right)}\;\mathrm{for}\;v_{t}=const$$ (4)

We rewrite this equation as

$$\frac{v\left(t\right)}{v_{t}}=\frac{2\left(v_{t}+v_{0}\right)}{v_{t}+v_{0}+\left(v_{t}-v_{0}\right)\exp\left(-2gt/v_{t}\right)}-1$$

By taking *v* = −*dh*/*dt* into account this equation can directly be integrated to deliver under the initial precondition $1/v_{t}^{2}=C_{D}\rho A_{⊥}/\left(2mg\right)=const$

$$h\left(t\right)=h_{0}+v_{t}t-\frac{v_{t}^{2}}{g}\ln\left\{\frac{1}{2}\left[1-\frac{v_{0}}{v_{t}}+\left(1+\frac{v_{0}}{v_{t}}\right)\exp\left(\frac{2gt}{v_{t}}\right)\right]\right\}$$ (5)

For a small initial time interval, *gt* ≪ *v*_*t*_, we derive after some lengthy approximation

$$h\left(t\right)=h_{0}-v_{0}t-\frac{1}{2}gt^{2}\left(1-\frac{v_{0}^{2}}{v_{t}^{2}}\right)\left[1-\frac{2}{3}\frac{v_{0}gt}{v_{t}^{2}}-\frac{1}{6}\left(1-3\frac{v_{0}^{2}}{v_{t}^{2}}\right){\left(\frac{gt}{v_{t}}\right)}^{2}-\;\ldots \right]$$

This simplifies for an initial zero speed *v*_0_ = 0 to the convenient expression

$$h\left(t\right)=h_{0}\underset{\mathrm{gravity}}{\underset{︸}{-\frac{1}{2}gt^{2}}}\underset{1\mathrm{st}\;\mathrm{order}\;\mathrm{drag}}{\underset{︸}{+\frac{1}{12}\frac{g^{3}}{v_{t}^{2}}t^{4}}}+\ldots$$

and

$$v\left(t\right)=-gt+\frac{1}{3}\frac{g^{3}}{v_{t}^{2}}t^{3}+\;\ldots$$

By the same token we now derive *v*(*h*). We first substitute

$$\frac{dv}{dt}=\frac{dv}{dh}\frac{dh}{dt}=-v\frac{dv}{dh}$$

In the equation of motion (3). We hence get

$$-v\frac{dv}{dh}=g\left(1-\frac{v^{2}}{v_{t}^{2}}\right)$$

and

$$-\int_{v_{0}}^{v}\frac{v\cdot dv}{1-v^{2}/v_{t}^{2}}=\frac{1}{2}v_{t}^{2}\ln\frac{v_{t}^{2}-v^{2}}{v_{t}^{2}-v_{0}^{2}}=g\int_{h_{0}}^{h}dh=g\left(h-h_{0}\right)$$

and finally

$$v\left(h\right)=v_{t}\sqrt{1-\left(1-\frac{v_{0}^{2}}{v_{t}^{2}}\right)\exp\left[\frac{2g}{v_{t}^{2}}\left(h-h_{0}\right)\right]}\;\mathrm{for}\;v_{t}=const$$ (6)

Eq (6) is a result of Mohazzabi and Shea [4] with generalized initial conditions.

### Analytical solution for *v*_*t*_ ≠ *const*

Yet, in reality *v*_*t*_ is not constant. In particular, and according to the barometric formula

$$\rho =\rho_{0}\exp\left(-\frac{h-h_{0}}{H}\right)$$

the atmospheric density decreases drastically with altitude and also somewhat with air temperature via the scale height

$$H\left(T\right)=\frac{TR_{s}}{g_{0}}=T\cdot 29.28\;m\;K^{-1}$$

where *R*_*s*_ = 286.91 *J kg*^−1^*K*^−1^ is the specific gas constant of standard atmosphere and *g*_0_ = 9.798 *m s*^−2^. With this we rewrite the equation of motion Eq (3) as

$$\frac{1}{g}\frac{dv}{dt}=1-\frac{v^{2}}{v_{t0}^{2}}\exp\left(-\frac{h\left(t\right)-h_{0}}{H}\right)$$ (7)

where

$$\frac{1}{v_{t0}^{2}}≔\frac{1}{2}C_{D}\rho_{0}\frac{A_{⊥}}{mg}$$ (8)

We note for later purposes that in free fall air drag always counteracts gravitational force and hence

$$\Delta v=g\cdot \Delta t-\int \frac{v^{2}}{v_{t0}^{2}}\exp\left(-\frac{h-h_{0}}{H}\right)\cdot dt<g\cdot \Delta t$$ (9)

Eq (7) can no longer be integrated fully analytically. However, for flight data analysis we only need to consider the change in velocity Δ*v* for small time intervals Δ*t*. In this case the analytical solution is given by the Taylor series

$$\Delta v={\dot{v}}_{0}\Delta t+{\ddot{v}}_{0}\frac{\Delta t^{2}}{2!}+\;\ldots$$

By Eq (7) we have not only $\dot{v}$, but with the approximation *h* = *h*_0_ − *v*_0_*t* we derive additionally

$$\frac{1}{g}\ddot{v}=-\left[\frac{2vv^{\prime }}{v_{t0}^{2}}+\frac{v^{2}}{v_{t0}^{2}}\frac{v_{0}}{H}\right]\exp\left(\frac{v_{0}t}{H}\right)$$

So, with definitions *t*_0_ = 0, *v*(*t*_0_) = *v*_0_ we obtain ${\dot{v}}_{0}$ and ${\ddot{v}}_{0}$, which inserted into the Taylor series delivers

$$\Delta v=g\left(1-\frac{v_{0}^{2}}{v_{t0}^{2}}\right)\cdot \Delta t-\frac{v_{0}g^{2}}{v_{t0}^{2}}\left[1-\frac{v_{0}^{2}}{v_{t0}^{2}}+\frac{v_{0}^{2}}{2gH}\right]\cdot \Delta t^{2}+\ldots$$ (10)

### Extraction of aerodynamic parameters from flight data

To derive aerodynamic parameters from flight data, we have to define a small-time interval Δ*t*, then read from the fight data the measured change in velocity Δ*v* in that interval, and with this finally solve Eq (10) for $v_{0}^{2}/v_{t0}^{2}$ to derive *C*_*D*_*A*_⊥_ from $v_{t0}^{2}$ via Eq (8). Because the Δ*t*^2^-term is much smaller than the Δ*t*-term, the Banach fixed-point theorem applies and we can solve Eq (10) for $v_{0}^{2}/v_{t0}^{2}$ iteratively by contraction mapping. So, in a first step we set the Δ*t*^2^-term to zero and find as a first approximation

$$\frac{v_{0}^{2}}{v_{t0}^{2}}\approx 1-\frac{\Delta v}{g\cdot \Delta t}>0$$

The latter follows from Eq (9). This is the trivial finding that attributes any deviation of constant acceleration from *g* to atmospheric drag. For a second-order approximation, we insert this result into the Δ*t*^2^-term of Eq (10), which yields

$$0=\Delta v-g\left(1-\frac{v_{0}^{2}}{v_{t0}^{2}}\right)\cdot \Delta t+\frac{g^{2}}{v_{0}}\left(1-\frac{\Delta v}{g\cdot \Delta t}\right)\left[\frac{\Delta v}{g\Delta t}+\frac{v_{0}^{2}}{2gH}\right]\cdot \Delta t^{2}$$

Again, solving for $v_{0}^{2}/v_{t0}^{2}$ delivers

$$\frac{v_{0}^{2}}{v_{t0}^{2}}=\left(1-\frac{\Delta v}{g\cdot \Delta t}\right)\left[1-\frac{\Delta v}{v_{0}}-\frac{v_{0}\Delta t}{2H}\right]$$ (11)

This is the second order solution of Eq (9) for a given Δ*t* and a measured Δ*v*. Analysis of the above derivation reveals that the Δ*v*/*v*_0_-term in square brackets accounts any variation of acceleration to an adjustment of the aerodynamic parameter *v*_*t*0_ from its trivial determination as given in the round brackets, and the *v*_0_Δ*t*/(2*H*)-term, which accounts for air density increase in the interval, makes an exception from this. We insert this advanced solution into Eq (8), and with the notation that the index 0 indicates values at the beginning of a time interval, we finally derive *C*_*D*_*A*_⊥_ for any small time interval

$$C_{D}A_{⊥}=\frac{2mg}{v_{0}^{2}\rho_{0}}\left(1-\frac{\Delta v}{g\cdot \Delta t}\right)\left(1-\frac{\Delta v}{v_{0}}-\frac{v_{0}\Delta t}{2H\left(h_{0}\right)}\right)$$ (12)

with

$$H\left(h_{0}\right)=T\left(h_{0}\right)\cdot 29.28\;\left[m\;K^{-1}\right]$$

### Transonic regime

The objective of our work is to understand the aerodynamic behavior of a human body with pressure suit in the transonic regime by deriving the drag coefficient *C*_*D*_ with Stratos’ flight data from Eq (12). The transonic regime is the velocity range around the speed of sound *a* or Mach number Ma = 1 with

$$Ma≔\frac{v}{a}=\frac{v}{\sqrt{\kappa_{air}R_{S}T}}$$ (13)

Here *κ*_*air*_ = 1.403 is the adiabatic index and *R*_*s*_ = 286.91 *J K*^−1^*kg*^−1^ is the specific gas constant of the standard atmosphere. Since Ma is temperature dependent (but not density-dependent as one might assume intuitively) an atmospheric temperature profile *T*(*h*) is essential to determine the correct Mach number.

## III. Data analysis procedure

For deriving the drag coefficient *C*_*D*_ from Eq (12) we need to have the following data

1. Flight trajectory data *v*(*t*) and *h*(*t*)
2. Atmospheric data, i.e. air density *ρ*(*h*) and air temperature data *T*(*h*).
3. Pressure suit data, i.e. the total mass m, incl. body weight, and the effective surfaces along his bodies symmetry axes *A*_*x*_,*A*_*y*_,*A*_*z*_
4. Flight attitude data, i.e. angle of attack *α*(*t*) of his body against flight direction, to derive the wetted area *A*_⊥_(*t*)

### Flight trajectory data

**Fig 1:**
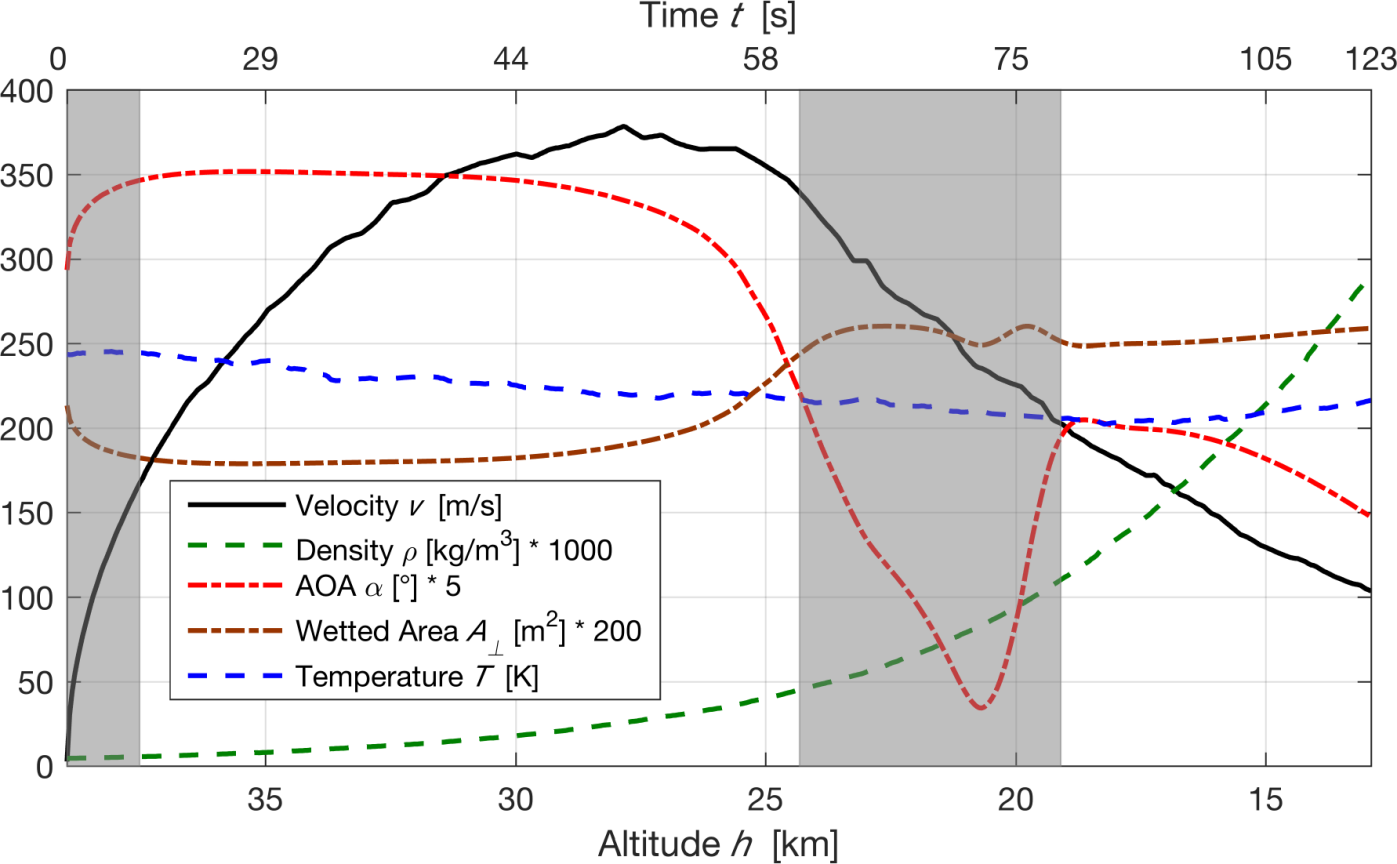
Summary of flight trajectory and atmospheric data. The gray area on the left indicates that for *t* < 17 *s*, owing to insignificant drag, no drag coefficient can be derived. The gray area on the right indicates times when Felix experienced a dangerous flat spin at a reduced AOA.

Felix jumped from the capsule at an altitude of 38969.4 m. At 50.0 *s* into flight and at an altitude of 28833 m he obtained his maximum vertical velocity of 377.1 m*s*^−1^ equaling Mach 1.25. His free fall lasted down to an altitude of 2566.8 m where he pulled the drag chute. The detailed flight trajectory data *v*(*t*) and *h*(*t*) were extracted from the graphical plots as given in the Summary Report of the Red Bull Stratos Team [9] by digitalization (see Fig 1). Velocity and time were measured by an on-body GPS system.

#### Atmospheric data

On October 12, at 4h 51min GMT, a radiosonde was launched from the airfield measuring the profile data *p*(*h*), *T*(*h*), wind direction, and wind speed. Based on this measurement a FIM forecast for flight day October 14 was generated. From this and with the ideal gas law, the atmospheric density profile was derived as

$$\rho \left(h\right)=\frac{p\left(h\right)}{R_{s}T\left(h\right)}$$ (14)

with *R*_*s*_ = 286.91 *J K*^−1^*kg*^−1^. Both profiles, *ρ*(*h*) and *T*(*h*), are depictured in Fig 1 with errors of about 0.1%.

### Flight attitude data and wetted area

**Fig 2:**
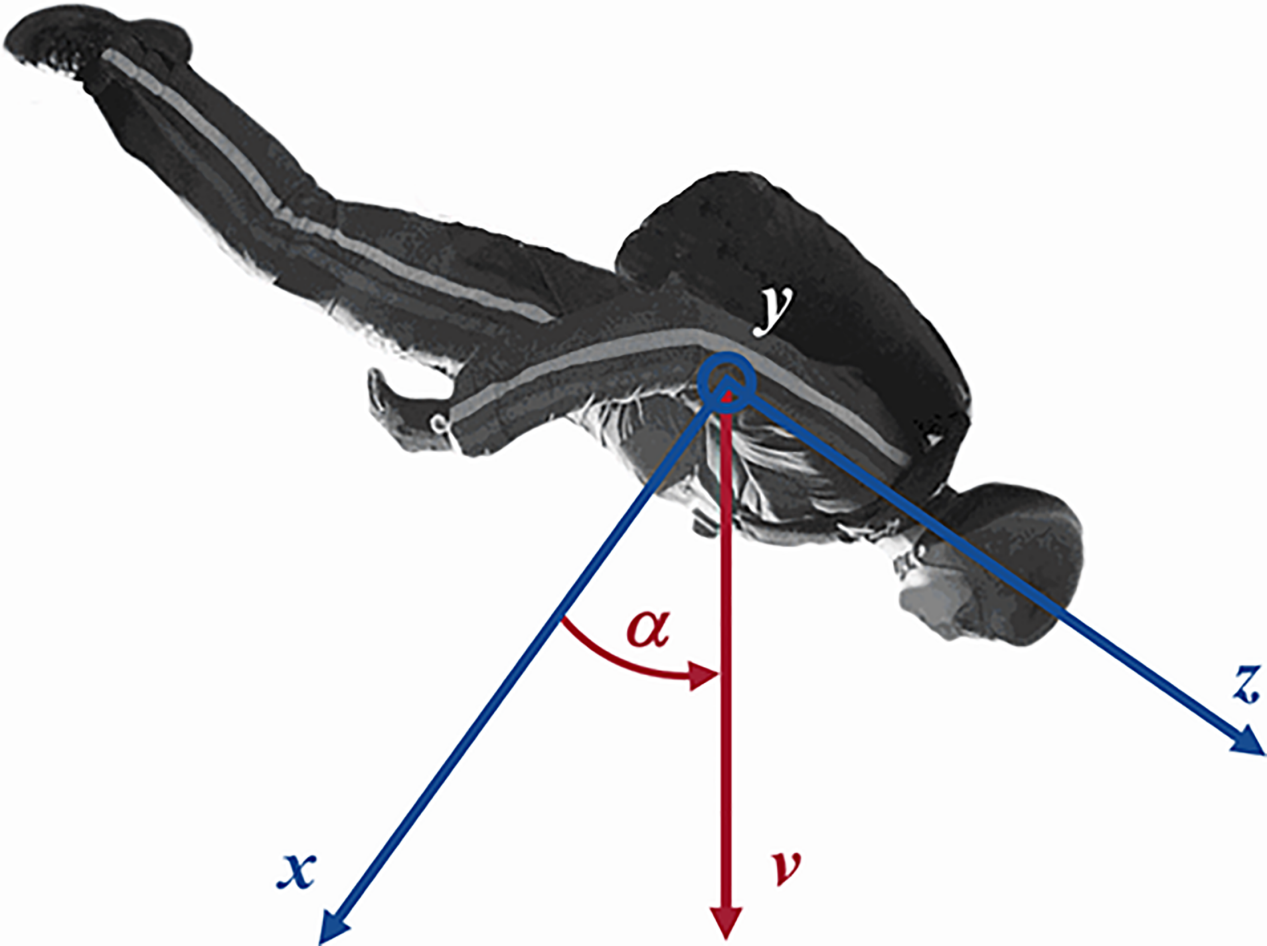
The body reference frame and definition of *α*(*AOA*).

The angle of attack (AOA), *α*(*t*), is defined (see Fig 2) such that *α* = 0 is a regular “belly down” attitude for skydivers. We derived *α*(*t*) (depicted in Fig 1) from the full story video published by GoPro [1]. The error of the AOA such determined is estimated to be 15–20%.

The wetted pressure suit surface area is determined from the AOA and roll angle *ϕ* (angle around the body *z*-axis) quite generally as

$$A_{⊥}=(A_{x}\cos\left(\phi \right)+A_{y}\sin\left(\phi \right))\cos\left(\alpha \right)+A_{z}\sin\left(\alpha \right)$$

Because Felix’ attitude after 17 seconds of free fall did not show any significant roll, i.e. *ϕ* = 0, we have

$$A_{⊥}=A_{x}\cos\alpha +A_{z}\sin\alpha$$ (15)

This confirms the expected result that for a belly-down attitude *A*_⊥_(*α* = 0) = *A*_*x*_.

### Pressure suit data

**Fig 3:**
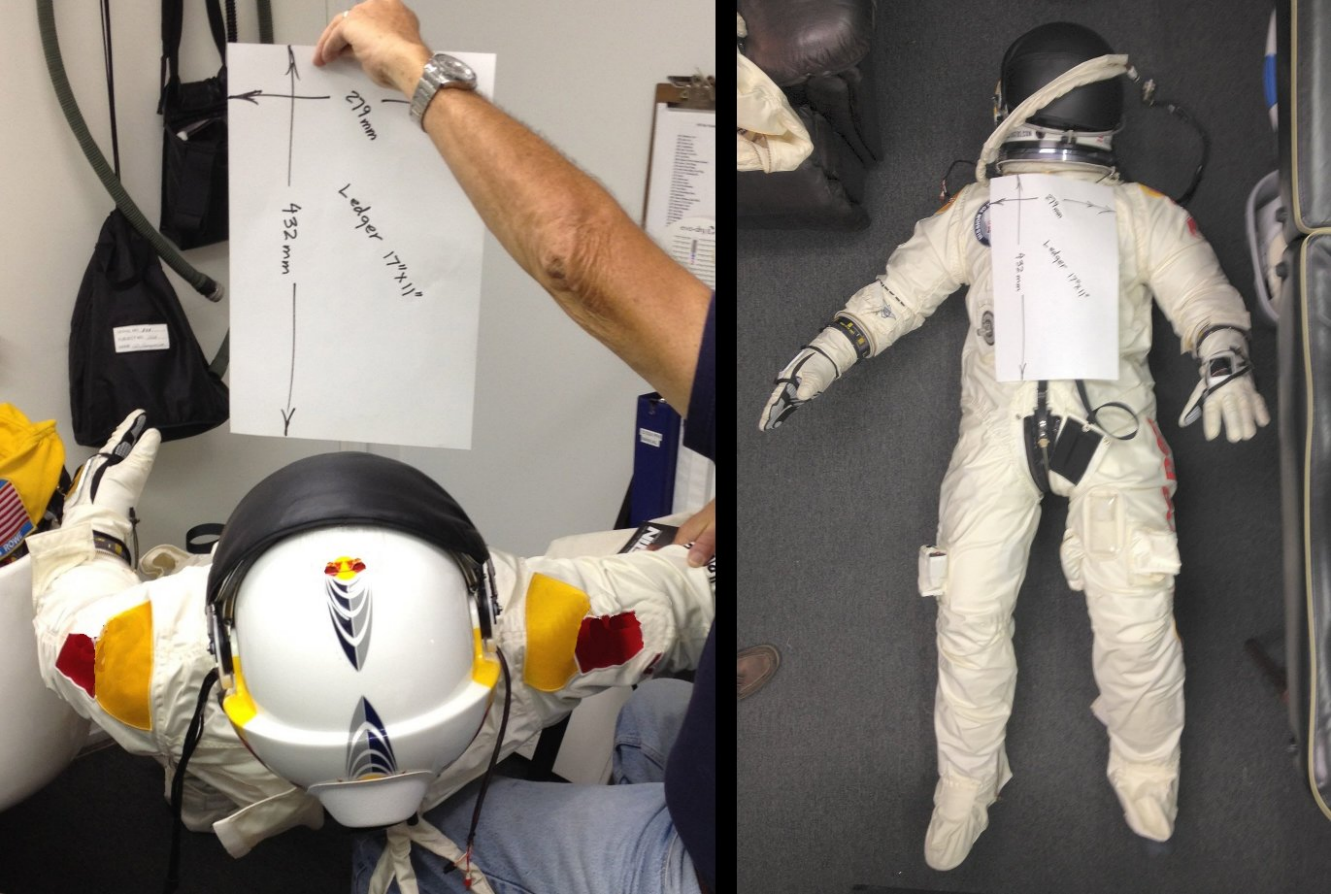
View of the pressure suit along the −*z*-axis (left) and −*x*-axis (right). The ledge is of size 17”x11” to determine its cross-section. (Credit: Art Thompson / Stratos team).

In order to determine the pressure suit data, on September 17, 2012, during a test run, pictures of the pressurized suit were taken (see Fig 3) along the three body frame axes as defined in Fig 2, always together with a sheet of paper of size 17″ × 11″ = 0.1206 *m*^2^ for area reference. From these the following effective suit cross sections were derived

$$\begin{array}{l} A_{x}=12.8\;ft^{2}=1.19\;m^{2}\\ A_{y}=8.6\;ft^{2}=0.804\;m^{2}\\ A_{z}=5.6\;ft^{2}=0.525\;m^{2} \end{array}$$ (16)

The total mass of Felix and the fully dress-up suit was determined to be

m=267.1lbs=121.2kg (17)

### Gravity of earth

For the gravity of Earth, we apply the WGS 84 ellipsoidal gravity formula in combination with a FAC altitude correction factor

$$g\left(h,\varphi \right)=9.780\frac{1+0.00193\cdot \sin^{2}\varphi }{\sqrt{1-0.00669\cdot \sin^{2}\varphi }}-3.086\times 10^{-6}\;\left[s^{-2}\right]\cdot h$$

with altitude *h* and geographical latitude *φ*. For *φ* = 33.39° at the jump site this reduces to

$$g\left(h\right)=9.796\;\left[m\;s^{-2}\right]-3.086\times 10^{-6}\;\left[s^{-2}\right]\cdot h$$ (18)

Hence, the altitude-dependent gravity introduces a relative *g* error of only 0.01%.

### Initial interval considerations

Eq (12) is applicable to flight data only if in the equation

$$\Delta v=g\cdot \Delta t-\frac{1}{2}C\rho \frac{A_{⊥}}{m}v^{2}\cdot \Delta t$$

the aerodynamic term is significantly larger than the velocity data error *δv*, i.e.

$$\frac{1}{2}C_{D}\rho \frac{A_{⊥}}{m}v^{2}\cdot \Delta t\geq \delta v$$

For our digitalization technique *δv* ≈ 0.05 ⋅ *v* and hence

$$\frac{1}{2}C_{D}\rho \frac{A_{⊥}}{m}v\cdot \Delta t\geq 0.05$$

This is particularly important for the initial free fall segment, where *ρv* ⋅ Δ*t* is extremely small. In this regime where Δ*t* = *t* and *v* ≈ *g* ⋅ *t* we get as the condition of the beginning *t*_0_ of the first relevant time interval

$$\frac{1}{2}C_{D}\rho \left(t_{0}\right)\frac{A_{⊥}}{m}gt_{0}^{2}=0.05$$

With the given pressure suit data and assuming an initial *C*_*D*_ = 0.65 and belly-down flight attitude, i.e. *A*_⊥_ = *A*_*x*_, we get

$$\rho \left(t_{0}\right)\;t_{0}^{2}=1.6\;kg\;s^{2}m^{-3}$$

Assuming $h_{0}\approx \frac{1}{2}gt_{0}^{2}$ and from the atmospheric density profile *ρ*(*h*), we finally get for the first data analysis interval

$$t_{0}=17\;s$$

### Sampling interval estimator

Because the contribution of the drag term to the velocity is strongly varying over altitude and hence time, we need to adapt the sampling interval width. It needs to be chosen such that the drag deceleration *δv* shall be minimum 3 times as much as the data imprecision equaling 1 *m*/*s*

δv≤−3m/s (19)

We chose Eq (10) for interval estimation. For that we assume $v_{0}^{2}/v_{t0}^{2}$ as given and specify Δ*v* = *δv*. With this, and Δ*h* ≈ *v*_0_ ⋅ Δ*t*, and discarding at high drag the gravitational term we obtain

$$\begin{array}{l} \delta v=-g\frac{v_{0}^{2}}{v_{t0}^{2}}\cdot \Delta t-\frac{v_{0}g^{2}}{v_{t0}^{2}}\left[1-\frac{v_{0}^{2}}{v_{t0}^{2}}+\frac{v_{0}^{2}}{2gH}\right]\cdot \Delta t^{2}+\;\ldots \\ \;=-g\cdot \Delta t\left[\frac{v_{0}^{2}}{v_{t0}^{2}}+\frac{gv_{0}}{v_{t0}^{2}}\left(1-\frac{v_{0}^{2}}{v_{t0}^{2}}+\frac{v_{0}^{2}}{2gH}\right)\Delta t\right] \end{array}$$

In first-order approximation this yields

$$\Delta t=-\frac{\delta v}{g}\frac{v_{t0}^{2}}{v_{0}^{2}}$$

In second-order approximation we have

$$\delta v=-g\cdot \Delta t\left[\frac{v_{0}^{2}}{v_{t0}^{2}}-\left(1-\frac{v_{0}^{2}}{v_{t0}^{2}}+\frac{v_{0}^{2}}{2gH}\right)\frac{\delta v}{v_{0}}\right]$$

And hence for time interval estimation

$$\Delta t=-\frac{\delta v}{g}/\left[\frac{v_{0}^{2}}{v_{t0}^{2}}-\left(1-\frac{v_{0}^{2}}{v_{t0}^{2}}+\frac{v_{0}^{2}}{2gH}\right)\frac{\delta v}{v_{0}^{}}\right]$$ (20)

with $v_{t0}^{2}$ given by Eq (8). If the time interval becomes so short that Δ*h* < 200 m we set Δ*h* = 200 m.

## IV. Results

### Analysis methods

To extract the aerodynamic parameter *C*_*D*_*A*_⊥_ from the data, we applied three different methods.

Method A is straightforward in that starting with a measured initial vertical speed it solves the equation of motion (1) and *C*_*D*_*A*_⊥_ is adjusted such that the vertical speed at the end of each time interval fits the measured value.

Method B makes use of the equation preceding Eq (6)

$$\frac{1}{2}v_{t}^{2}\ln\frac{v_{t}^{2}-v^{2}}{v_{t}^{2}-v_{0}^{2}}=g\left(h-h_{0}\right)$$

For a given set of measured interval data *h*_0_,*v*_0_;*h*,*v* this equation is solved for *v*_*t*_ by the trust-region method with dogleg step strategy. We recall that this method B assumes that *v*_*t*_ and hence the atmospheric density *ρ* is constant over each interval.

Method C makes use of Eq (12), which is explicit in *C*_*D*_*A*_⊥_ and in addition takes into account also linear variations of air density and scale height variations over each interval.

Overall, the results of all three methods did not show any grave qualitative differences. However, because method A and method B featured some numerical instabilities in particular at high altitudes while method C was not only stable throughout the data range, but is also physically more elaborated, we discuss here only the results derived by method C. For a detailed comparison of the three methods see section *Sensitivity Analysis–Different Methods* below.

Method C is based on the approximation *v*⋅Δ*t* = Δ*h* ≪ 2*H* ≈ 14 km. A lower Δ*h*-limit arises from increasing relative velocity gain errors with decreasing Δ*h*. We hence settled at an interval width of Δ*h* = 200 m. According to the above section *Initial Interval Considerations*, the first interval started at *t*_0_ = 17 *s* into flight corresponding to an altitude of *h*_0_ = 37 640 m. Thereafter we used for the time interval estimator, Eq (20), with *δv* = −10 *m*/*s* down to *h*_*end*_ = 13040 m.

**Fig 4:**
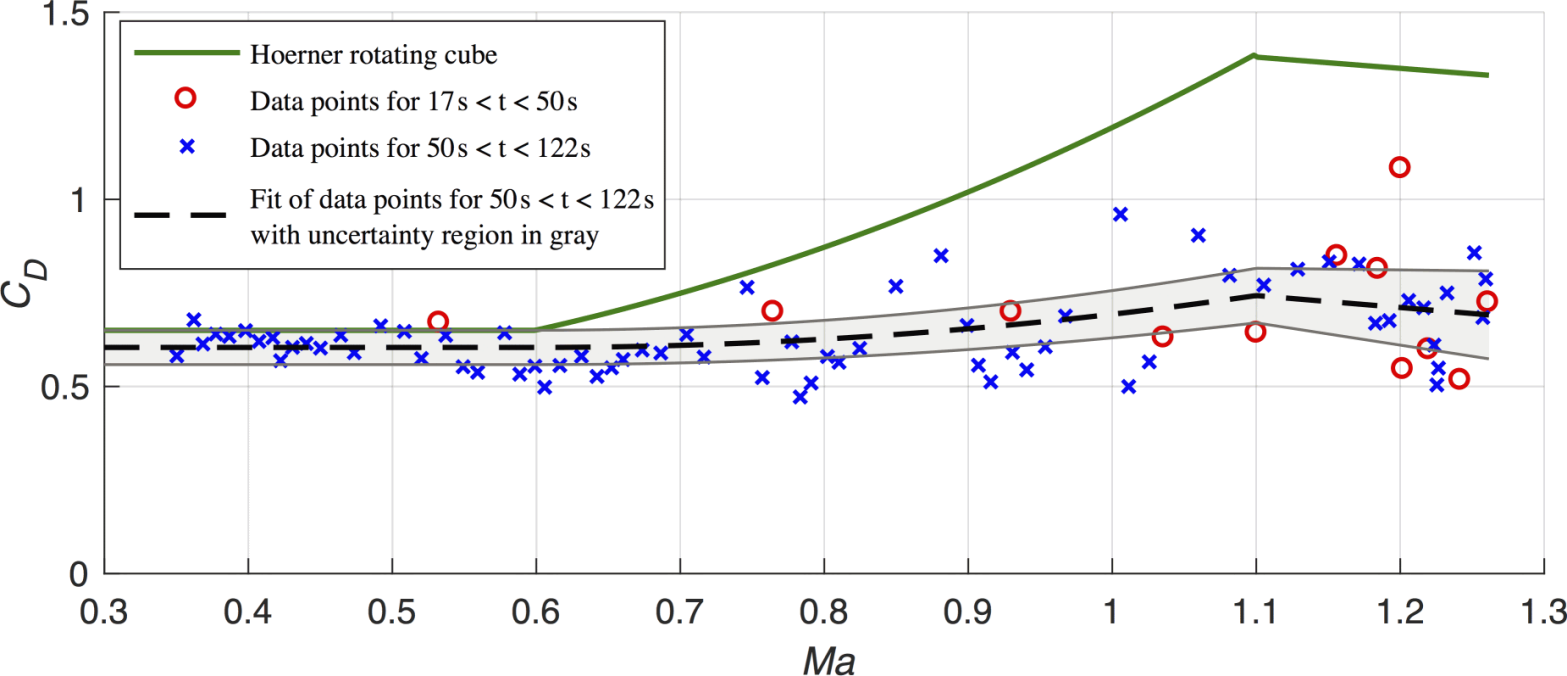
Drag coefficient as a function of Mach number. Red circles for increasing and blue crosses for decreasing velocities. The dashed line is the final result as given by Eq (26) and the gray region the total uncertainty as given by Eq (25). The green line is the theoretical transonic drag coefficient for a rotating cube according to Hoerner [11] and as given by Eq (21).

Fig 4 displays the resulting aerodynamic drag coefficient as a function of Mach number. We note that the absolute inaccuracy of the data is mainly driven by the inaccuracy of the area A(AOA). Therefore, we performed a sensitivity analysis described in Section V.

There are two distinct phases (cf. full line in Fig 1): A velocity increase phase for 17*s* < *t* < 50*s* (red circles) and a velocity decrease phase 50 *s* < *t* < 120 *s* (blue crosses). In addition, a green line is shown, which according to Hoerner [11] is the transonic drag coefficient for a rotating cube, namely

$$\begin{array}{l} C_{D}=0.65\;\mathrm{for}\;Ma\leq 0.6\\ C_{D}=0.50+1.23{\left(Ma-0.25\right)}^{2}\;\mathrm{for}\;0.6\leq Ma\leq 1.1\\ C_{D}=1.38-0.30\left(Ma-1.1\right)\;\mathrm{for}\;1.1\leq Ma \end{array}$$ (21)

This empirical dependency was used by the Red Bull team to predict the maximum velocity at a given exit altitude, or the required altitude to ensure that his maximum speed would achieve Mach 1.15 vice versa and hence achieve supersonic speed of a free falling human body for the first time.

The scatter of data point and equivalently the data error obviously is much bigger for the increasing velocity data points than for the decreasing velocity data points. This is because the drag *D* ∝ *ρv*^2^ affects the measured speed with decreasing altitude and increasing speed. Nevertheless, the Mach dependency of the up-velocity and down-velocity data points coincide. We therefore now focus on the more accurate down-velocity data (blue crosses).

To derive an empirical drag coefficient dependence on the Mach number, we use the same intervals and dependencies as Eq (21), but with the three variable fit parameters A, B, and C:

$$\begin{array}{l} C_{D}=A\;\mathrm{for}\;Ma\leq 0.6\\ C_{D}=A+B{\left(Ma-0.6\right)}^{2}\;\mathrm{for}\;0.6\leq Ma\leq 1.1\\ C_{D}=A+B\cdot 0.25-C\left(Ma-1.1\right)\;\mathrm{for}\;1.1\leq Ma\leq 1.25 \end{array}$$ (22)

From fitting the downstream data, we derive the fit parameter as

$$\begin{array}{l} A=0.60\pm 0.05\\ B=0.55\pm 0.10\\ C=0.32\pm 0.10 \end{array}$$ (23)

## V. Sensitivity analysis

### Different methods

**Fig 5:**
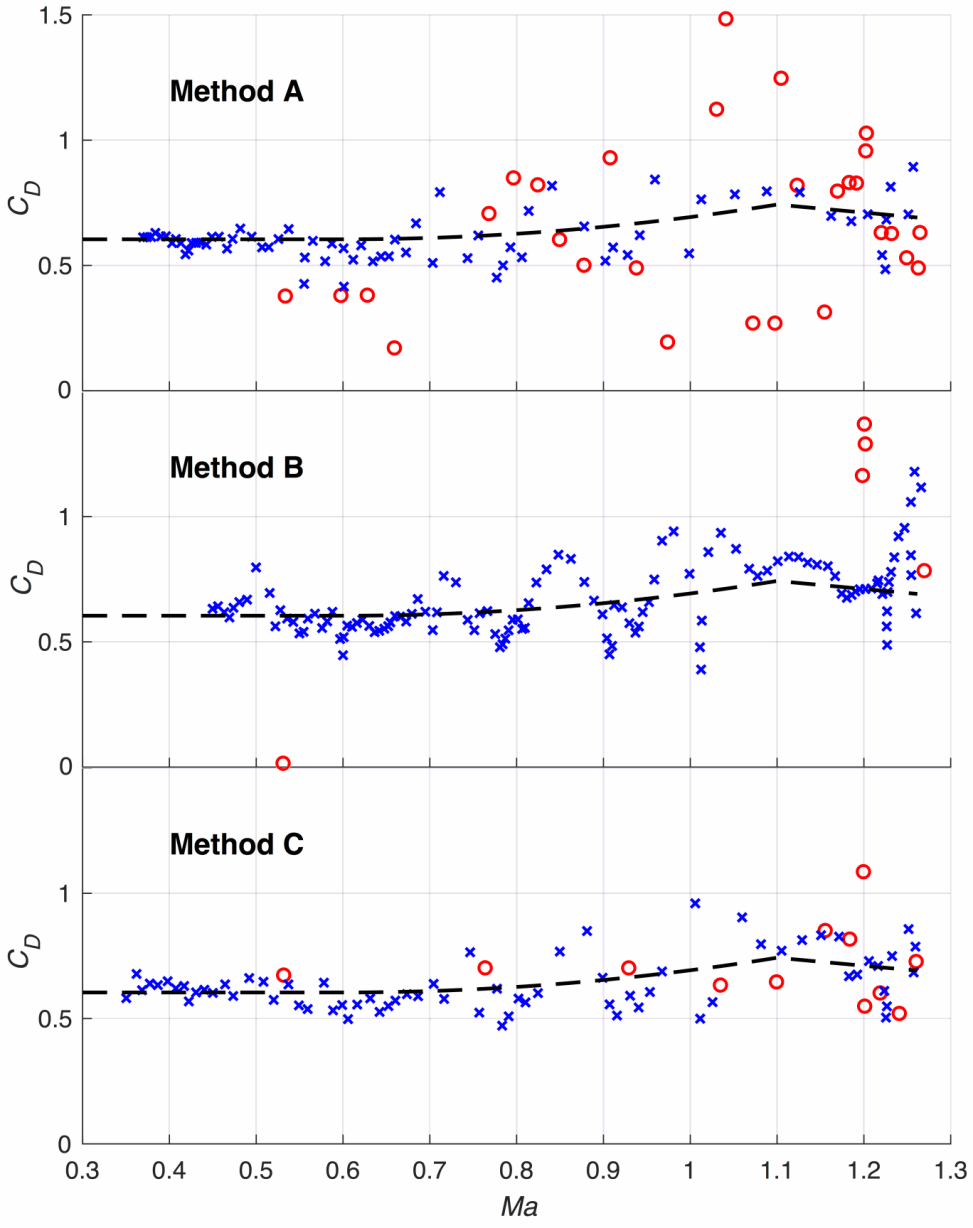
The drag coefficient as derived from the three different methods A, B, and C.

In Fig 5 the drag coefficients are provided as derived from the three different methods A, B, C (see Section *Results –Analysis Methods* above). In all three cases, the dashed line is the data function as given by Eqs (22) with (23), i.e. as derived from method C. The data shows that although all three methods provide qualitatively the same results, method C obviously delivers the best result.

### Initial interval width

**Fig 6:**
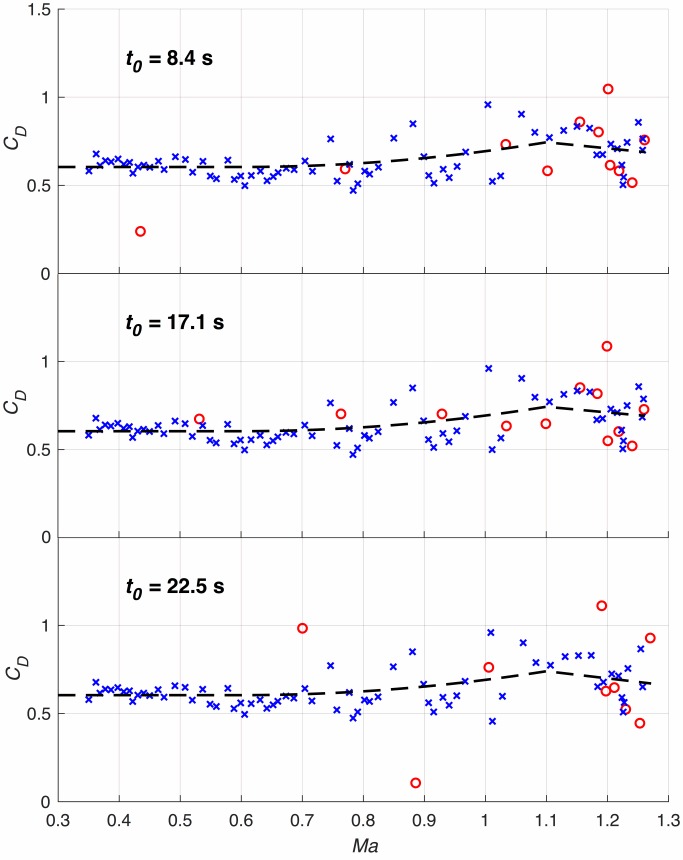
The drag coefficient as derived with different initial interval widths. ***t***_**0**_ **= 17.1 *s*** was finally chosen from theoretical considerations.

Next, we study the effect of the width of the omitted initial interval (see section Data Analysis Procedure *–Initial Interval Considerations*) on the results. As shown in Fig 6 we have varied the initial interval in a reasonable range. Besides minor differences, the data points are qualitatively the same (particularly the downstream data point, to which Eq (22) was fitted).

### Sampling interval width

**Fig 7:**
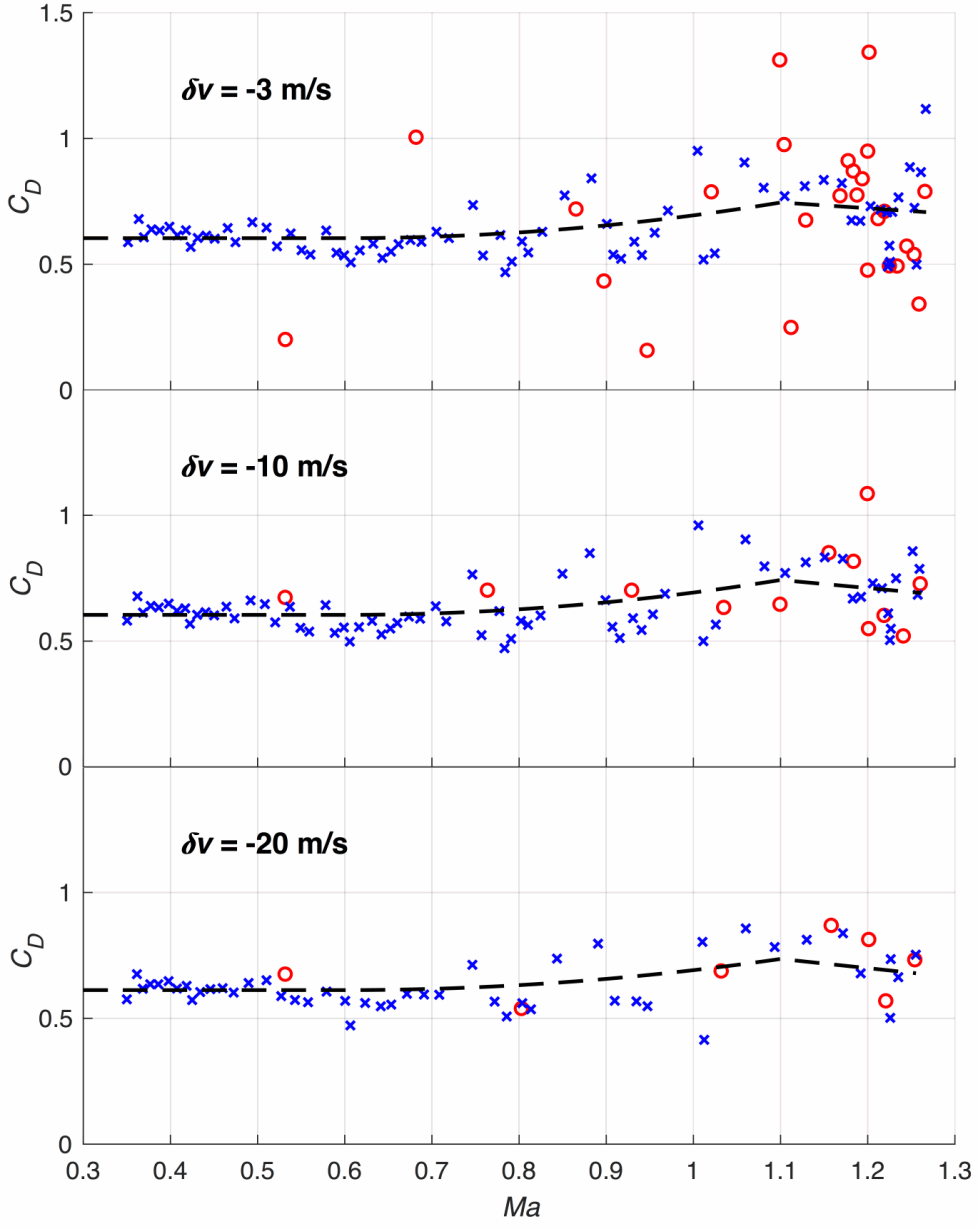
The drag coefficient as derived from different sampling time intervals. Intervals are given by the different speed changes *δv* within the interval (see Eq (20)).

The sampling interval is expected to have more effect on the results than all the other parameters. Fig 7 shows the data points for three different velocity increase intervals *δv*. According to Eq (20) a changing *δv* results in different time intervals. Obviously, the choice *δv* = −10*m*/*s* from theoretical considerations seems optimal.

### AOA uncertainty

**Fig 8:**
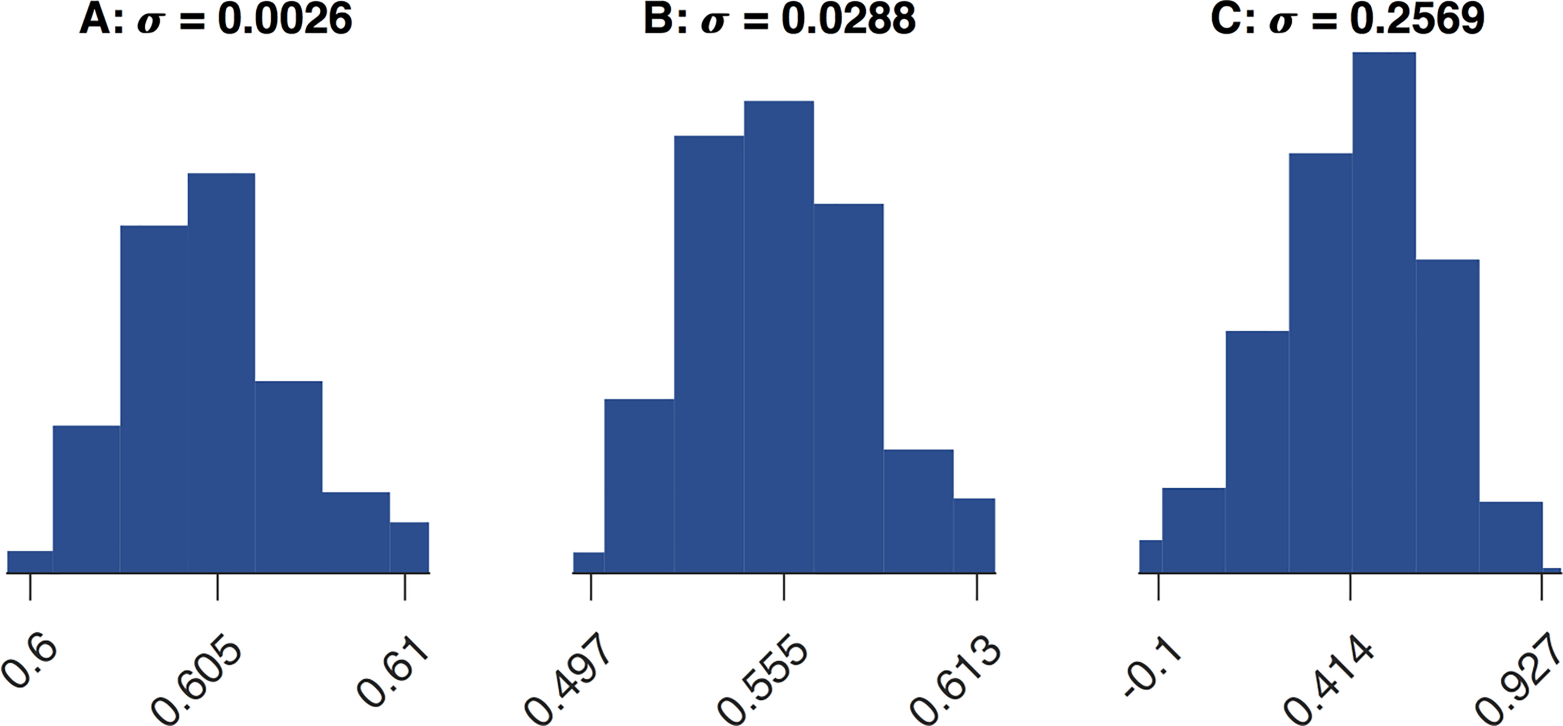
**AOA sensitivity analysis for fit parameters A, B, and C.** For each trajectory time step the AOA was randomly sampled 1000 times with variations up to 20%. The abscissae show the value of a fitting parameter binned in seven value ranges. The vertical bar to each range measures the number of values obtained from the variations that fall into this range. From the resulting histogram, the standard variation σ for each of the fitting parameters A, B, and C is derived.

To study the impact of the relatively high AOA uncertainty on the resulting drag coefficient as given by Eq (22) with Eq (23), we performed a sensitivity analysis by randomly varying the AOA for each time step by up to 20% around its value shown by in Fig 1 (AOA limited to 0° and 90°). We ran 1000 samples and calculated for each value the fitting parameters A, B, and C. A histogram of them is shown in Fig 8 with the standard variation σ. The value of the fitting parameter is depicted on the abscissae and their quantity on the ordinate. The gray area in Fig 4 shows the ±*σ* uncertainty of the distribution function. It can be seen from the error in Eqs (24) that for increasing Mach numbers the error due to the AOA uncertainty increases, in particular for the slope C of the line for *Ma* > 1.1, because it shows with *σ* = 0.26 the highest variation. For the derived ±σ the corresponding error of the fitting parameters are

$$\begin{array}{l} A=0.60\pm 0.003\\ B=0.55\pm 0.03\\ C=0.32\pm 0.26 \end{array}$$ (24)

Note, that the mean of the sensitivity analysis matches our calculated fitting parameters for A and B. For the slope C they do not match exactly. This is due to the limitation of the AOA to 0° and 90° and the high sensitivity of this fitting parameter.

The combined error of the data statistics Eq (23) and the AOA sensitivity analysis Eq (24) is

$$\begin{array}{l} A=0.60\pm 0.05\\ B=0.55\pm 0.11\\ C=0.32\pm 0.28 \end{array}$$ (25)

The final result reads

$$\begin{array}{l} C_{D}=0.60\;\mathrm{for}\;Ma\leq 0.6\\ C_{D}=0.60+0.55{\left(Ma-0.6\right)}^{2}\;\mathrm{for}\;0.6\leq Ma\leq 1.1\\ C_{D}=0.74-0.32\left(Ma-1.1\right)\;\mathrm{for}\;1.1\leq Ma\leq 1.25 \end{array}$$ (26)

This continuous function together with the total error as given by Eq (25) is displayed in Fig 4 as a dashed line and the gray region.

Compared to theoretical expectations for subsonic speeds as given by Eq (21) we have with *C*_*D*_ = 0.60 ± 0.05 a perfect match between our data and theoretical expectations. In addition, our overall pattern, is compliant with an increase of *C*_*D*_ below the sonic speed and a decrease above it. However, for transonic speeds we see an increase of the drag coefficient of just Δ*C*_*D*_ = 0.14, which is only 19% of the theoretically expected Δ*C*_*D*_ = 0.74.

## VI. Discussion

**Fig 9:**
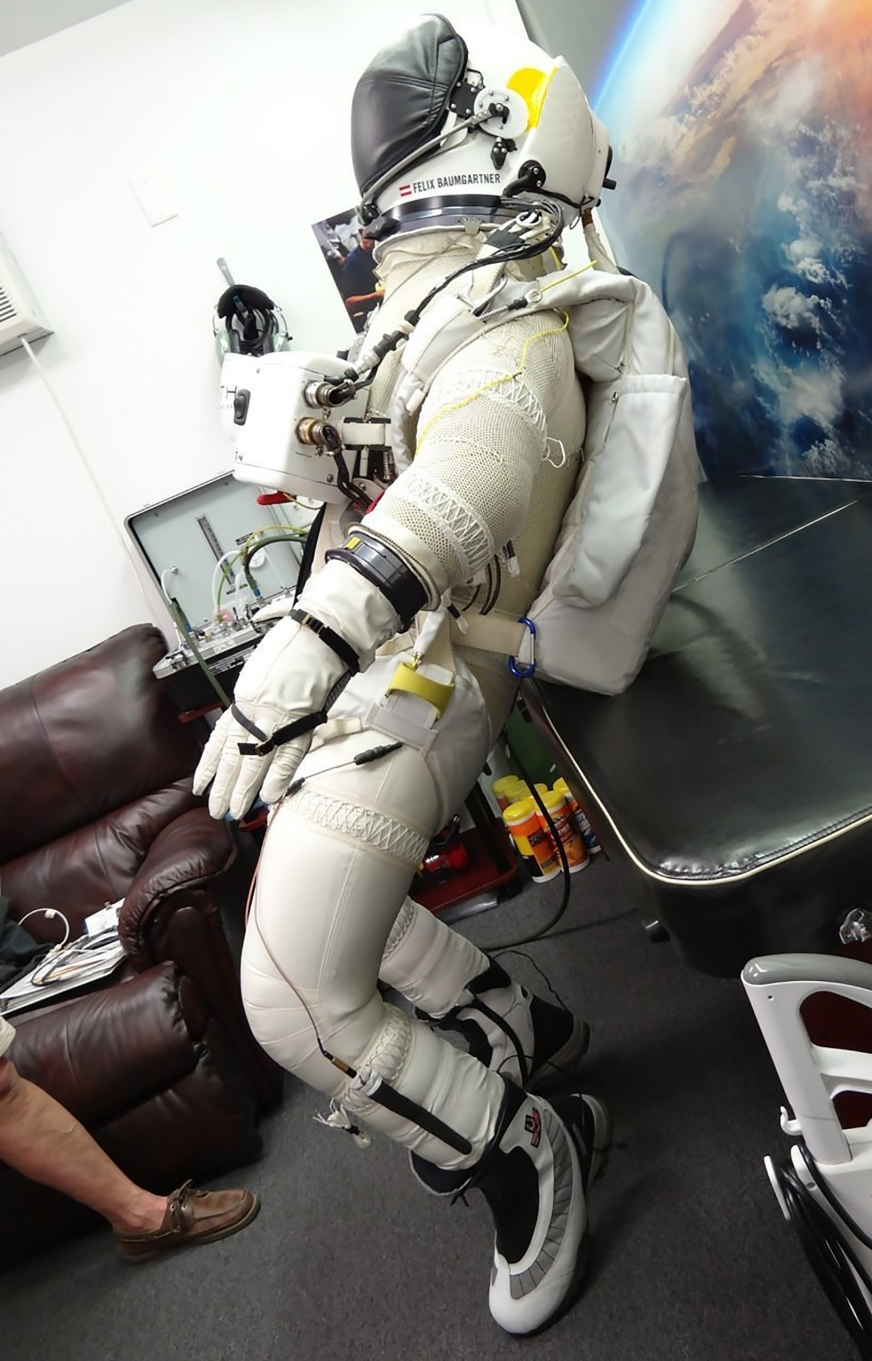
The complex suit shape evoking an equally complex aerodynamics. (Credit: Art Thompson / Stratos team).

In order to grasp this unexpected result, we apply fluid dynamics theory (Standard literature for further information on aerodynamics can be found in reference [10] and [11].). The key elements are the overall shape of the body and the relevant Reynold numbers. Relative to the wind flow, Baumgartner’s body is quite blunt, where the pressure suit with its folds adds roughness, while the technical equipment and cameras cause an unevenness of the surface (see Fig 9). Moreover, the backpack and Baumgartner’s limbs form even a quite complex body shape. We therefore identify Baumgartner’s irregular blunt body very approximately by a cylinder with surface roughness on all length scales.

**Fig 10:**
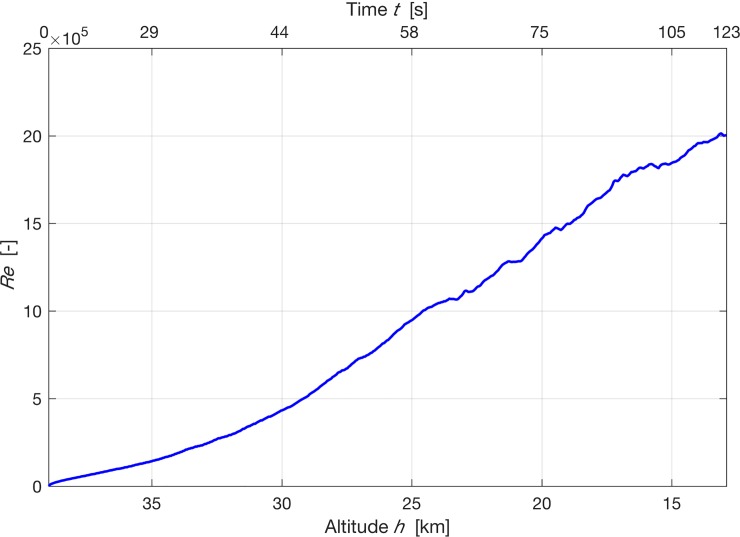
Reynolds number as a function of time into flight. The representative physical length scale of 1 *m* and a constant dynamic viscosity of 15×10^−6^ *kg s*^−1^ *m*^−1^.

The Reynolds number *Re* (see Fig 10) describes the proportion between the inertial and viscous forces and hence the type of flow, laminar or turbulent. It is between 2.3 ⋅ 10^5^ − 12.2 ⋅ 10^5^ for the considered time zone 29 s < t < 68 s.

### Drag crisis

**Fig 11:**
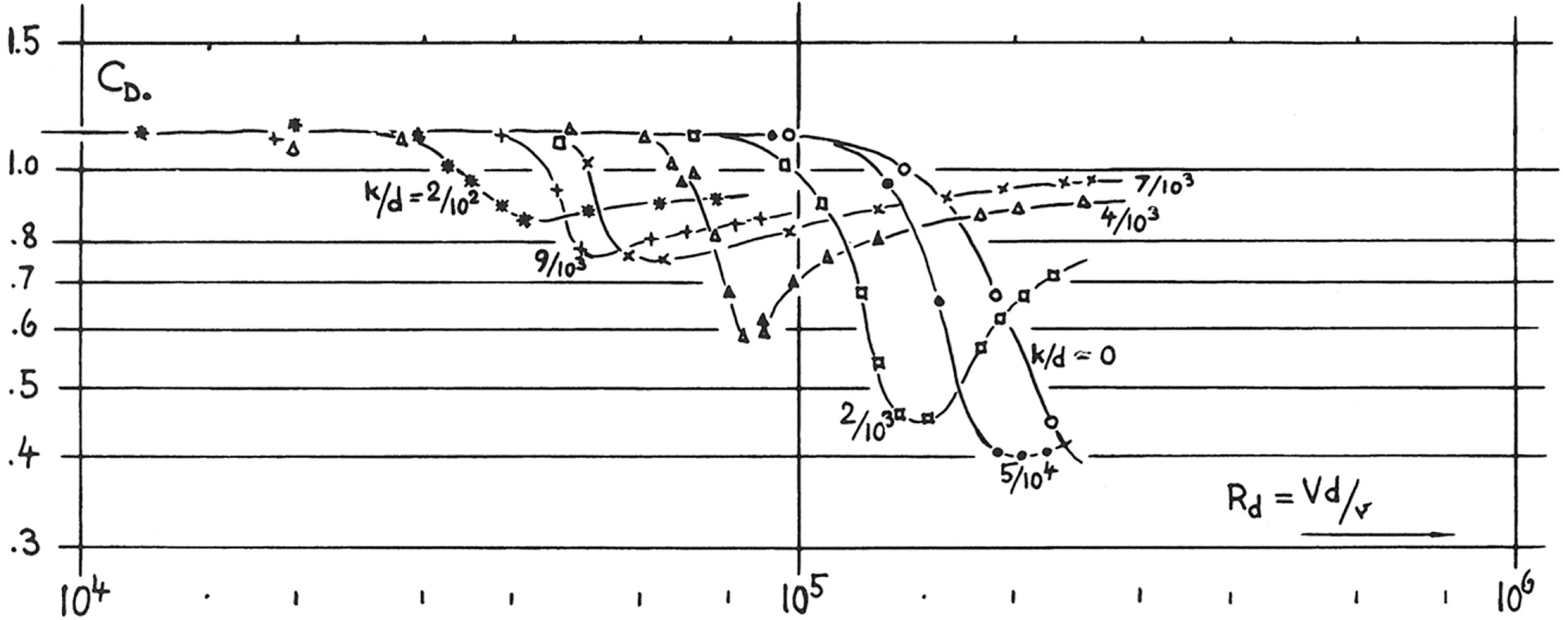
Drag crisis of cylinders as a function of surface sand-grain roughness k against cylinder diameter d. (from S.F. Hoerner [11]).

Fluid dynamic tells us that blunt bodies, i.e. bodies which are not streamlined, are subject dominantly to pressure drag and only little to skin friction drag. Because they are blunt they undergo a so-called *drag crisis* (see Fig 11) for *Re* ≈ 10^5^. Drag crisis is a drop of drag by up to a factor of 10 for smooth surfaces like a table tennis ball. The drop is due to the fact that the laminar flow from the stagnation point to the separation point, located at maximum body cross-section) becomes turbulent, which moves the separation point of the flow backward (delayed separation). This reduces recirculation behind the blunt body and hence lowers drag. However, if the surface is rough (sand-grain size k increases), turbulence in the upstream flow is induced already at lower Reynolds number, causing a less pronounced drag drop (see Fig 11). If the surface is very rough, uneven, and the body’s shape is even not well defined, as in our case, we do not expect any drag crisis because we have turbulence all over the surface at any Reynolds numbers *Re* > 10^4^. We therefore do not expect, and in fact do not see, any dependency of *C*_*D*_ from the Reynolds number below the critical Mach number. In our analysis, the constant *A* in Eq (22) models this constant pressure drag.

### Wave drag

At the critical Mach number local supersonic flow and hence shock waves start to emerge from major surface discontinuities (edges) where the flow is forced to accelerate. This erratic shock formation and general flow instabilities correspond to pressure discontinuities, which transform a considerable part of the impinging flow energy into heat. This loss in energy corresponds to a counteracting force, the so-called wave drag (a.k.a. transonic compressibility drag). However, for Mach number up to 0.7 or 0.8 compressibility effects have only minor effects on the flow pattern and drag. For increasing Mach numbers the surface extent that generates shock wave increases and the shock waves intensify. They now interact with the boundary layer so that a separation of the boundary layer occurs immediately behind the shock. This condition accounts for a large increase in drag, which is known as shock-induced (boundary-layer) separation. This separation empirically causes a roughly quadratic increase of the wave-drag coefficient with Mach number. Generally, the term *B*(*Ma* − 0.6)^2^ in Eq (22) models the wave drag.

Once a supersonic flow has been established, however, the flow stabilizes and the drag coefficient is reduced. We then essentially have a shock wave that builds up at the upstream front of the body and another one at the unsteady geometry at the downstream tail of the body. It is usually modeled by the term – *C*(*Ma* − 1.1) in Eq (22). The shock waves generate the characteristic double boom of a supersonic body flying past, which was indeed captured for Baumgartner’s supersonic free fall in this video recording provided here http://www.youtube.com/watch?v=yZFz6y4UCuo.

Given this fluid dynamics effects it becomes clear why drag crisis and the wave-drag coefficient is so insignificant: Because the body is quite blunt, the pressure drag seems to be dominant all the way up to sonic speed. The surface roughness and the complex body shape at all speeds seem to induce a highly turbulent flow even close to the surface. This destroys any boundary layer, thus strongly suppressing a delayed flow separation effect and shock-induced boundary-layer separation.

## Abbreviations

*A*_⊥_: wetted pressure suit surface area
C_D_: drag coefficient
g: Earth’s gravity
m: mass
Ma: Mach number
Re: Reynolds number
R_S_: specific gas constant
t: time
T: temperature
v: velocity
h: altitude (height)
α: angle of attack (AOA)
φ: degree of latitude
